# Intestinal parasitosis and anaemia among patients in a Health Center, North Ethiopia

**DOI:** 10.1186/s13104-017-2957-2

**Published:** 2017-11-28

**Authors:** Megbaru Alemu, Birhane Kinfe, Desalegn Tadesse, Wondemagegn Mulu, Tadesse Hailu, Endalew Yizengaw

**Affiliations:** 1 Department of Microbiology, Immunology and Parasitology, Bahir Dar University, PO Box 79, Bahir Dar, Ethiopia; 2 Adwa Health Center, Tigray, Ethiopia; 3 Department of Medical Parasitology and Vector Biology, Mekelle University, Mekelle, Ethiopia

**Keywords:** Intestinal parasite, Anaemia, Adwa, Ethiopia

## Abstract

**Objective:**

The aim of this cross-sectional study was to determine the magnitude of intestinal parasitosis and anaemia in a Health Center, North Ethiopia.

**Results:**

A total of 427 outpatients were enrolled and the median age of the participants was 22 years. The prevalence of intestinal parasitosis was 143 (33.5%). Age, place of residence and occupation were significantly associated with intestinal parasitosis. When we see parasite specific factors, significant associations were observed for source of drinking water (P = 0.02), age (P < 0.001) and family size (P = 0.003), respectively with *Entameba histolytica*, Hookworm and *Giardia lamblia* infections. The overall prevalence of anaemia was 35 (8.2%). The mean haemoglobin concentration among the study participants was 12.8 mg/dl. The highest prevalence of anemia was recorded for the age group of 15–19 years (29.6%). The proportion of anemia among intestinal parasite -infected and non-infected participants was 10.7 and 7.0%, respectively. Study participants infected with *S. stercoralis* and hookworm were more likely to develop anaemia than the non- infected ones; AOR (adjusted odds ratio) = 5.3, 95% CI (1.01–27.4); P = 0.028 and AOR = 11.1, 95% CI (3.36–36.9); P = 0.000, respectively.

**Electronic supplementary material:**

The online version of this article (10.1186/s13104-017-2957-2) contains supplementary material, which is available to authorized users.

## Introduction

Different species of intestinal parasites are responsible for majority of human infections resulting in considerable morbidity and mortality worldwide [1]. Roughly, a quarter of the world’s population is infected by one or other species of intestinal parasites [2]. Prevalence of intestinal parasitic infections varies in person, time, age and sex [3]. In Ethiopia, different studies addressed the magnitude of intestinal parasitosis among school-age children [4–12]. However, the pattern of intestinal parasitism in a population with diverse groups of people was not illustrated.

In addition to the wide-range morbidity and mortality, intestinal parasitosis (IP) is also associated with malnutrition, anaemia, impaired mental function, impaired verbal ability, physical weakness and low educational achievement in school children [13, 14]. For instance, hookworm infection cause anaemia by increasing blood and iron loss in the intestinal tract [15]. Some other species such as *Ascaris lumbricoides, Entamoeba histolytica, Trichuris trichiura, Strongyloides stercoralis* and *Giardia lamblia* have also been found to have great effect on nutritional status due to increased metabolic rate, anorexia and diarrhoea [15, 16]. IPs cause decreased intake or a functional increase in the body’s nutrient requirement by their interference with absorptive surfaces, physical obstruction of intestinal lumen, production of proteolytic substances, and consumption of nutrients intended for the body [14, 17, 18].

Only few studies have been conducted describing the possible contribution of intestinal parasitic infection for occurrence of anaemia in a community in Ethiopia in general and the current study area in particular. Hence, investigations focusing on this issue are particularly appropriate for the design of integrated control strategies aimed at reducing anaemia and IPs, including anti-parasitic treatment programs and micronutrient supplementation in a community basis. In light of this, the present study was aimed at determining the magnitude of IP and examining the relationships of haemoglobin (Hb) concentration and anaemia with IPs among outpatients at Adwa Health Center, North Ethiopia.

## Main text

### Methods

#### Study design, period and area

A cross-sectional study was conducted from February to June, 2016 at Adwa health centre. Adwa is found in the central zone of Tigray regional state, 946.1 km away from Addis Ababa. It is found in the longitude and latitude of 14°10′N 38°54′E and an elevation of 1907 m above sea level. Adwa Health Center receives patients from the town and the surrounding districts.

#### Study participants

The study participants for this particular research work were volunteered patients 15 years and above who were requested for stool examination in Adwa Health Center during the study period. Study participants who took medication for IPs within 1 month prior to the study were excluded. The study participants were selected by a non-probability convenience sampling.

#### Study size

A sample size of 427 was determined using single population proportion statistical formula by the following assumptions: 95% level of confidence, 5% margin of error and P (proportion) of 0.5, and non-response rate of 10%.

#### Data collection

*Questionnaire*: Data on demographic characteristics of study participants and associated factors of IP were collected using structured and pre-tested questionnaire via face-to-face interview. The interview was conducted by trained nurses.

*Stool examination*: The patients were supplied with labeled stool containers with tight covers bearing serial numbers of the subjects. The entire stool samples were received at the spot at an organized central place.

Faecal specimens were processed by wet mount and Kato-Katz techniques. Microscopic examination was made by experienced technicians under 10× and 40× objective lenses.

*Haemoglobin estimation*: Five milliliter of whole blood was collected into EDTA tubes through vein puncture from study participants. The level of haemoglobin was determined using Drabkin’s Cyanmethemoglobin method. Initially, the whole blood was diluted in Drabkin’s solution which lyses RBCs and release Hb into the solution. Oxidation of ferrous ions by potassium ferricyanide to ferric ions results in the formation of methemoglobin. Methemoglobin combines with the cyanide ions (CN-) forming Cyanmethemoglobin and absorbance is measured spectrophotometrically at 540 nm [40].

#### Quality control

Questionnaires were pre-tested on a small number of adults other than the study participants. The collected data were checked daily for consistency and accuracy. Standardized procedures were strictly followed during collection of stool and blood samples and analytical processes.

#### Statistical analysis

Data were entered and analyzed using statistical software package (IBM Comp. released2011. IBM SPSS statistics for windows, version 20 Armonk, NY: IBM comp.). Descriptive statistics were used to describe study participants in relation to relevant variables. Chi square and logistic regression tests were employed to measure association of dependent and independent variables. Those variables with P < 0.2 in the binary logistic regression were taken to multiple regression analysis and the AOR was calculated to control potential confounders. P-values less than 0.05 were taken statistical significant.

### Results

#### Demographic characteristics

A total of 427 study participants were enrolled in the current study. Of them, 50.1% were females. The median age of participants was 22 years. The mean family size was 3.7. Most were government employees (39.8%) followed by farmers (36.5%). Regarding educational status, 67.4% had completed secondary schools and 24.6% attended their tertiary educations.

Pipe-borne water was the main water source for drinking (81.5%) followed by spring water (15.2%). Most (73.8%) were urban residents (Table 1).

**Table 1:** Prevalence of intestinal parasitosis among patients at Adwa Health, North Ethiopia 2016

	Intestinal parasitosis		Total (%)	X^2^; P-value
	Positive N (%)	Negative N (%)		
Age (years)				
15–19	67 (41.9)	93 (58.1)	160 (37.5)	11.4; 0.03
20–24	30 (27.5)	79 (72.5)	109 (25.5)	
25–29	16 (32.7)	33 (67.3)	49 (11.5)	
30–34	10 (35.7)	18 (64.3)	28 (6.5)	
35–39	5 (23.8)	16 (76.2)	21 (4.9)	
> = 40	15 (25.0)	45 (75.0)	60 (14.1)	
Total	143 (33.5)	284 (6.5)	427 (100)	
Gender				
Male	76 (35.7)	137 (64.3)	213 (49.9)	1.76; 0.59
Female	67 (31.3)	147 (68.7)	214 (50.1)	
Total	143 (33.5)	284 (6.5)	427 (100)	
Source of drinking water				
Spring	27 (41.5)	38 (58.5)	65 (15.2)	2.6; 0.61
Pipe	109 (31.3)	239 (68.7)	348 (81.5)	
Well dug	7 (50.0)	7 (50.0)	14 (3.3)	
Total	143 (33.5)	284 (66.5)	427 (100)	
Occupation				
Civil servant	49 (28.8)	121 (71.2)	170 (39.8)	7.8; 0.01
Trader	20 (33.3)	40 (66.7)	60 (14.1)	
Farmer	64 (41.0)	92 (59.0)	156 (36.5)	
Housewife	10 (24.4)	31 (75.6)	41 (9.6)	
Total	143 (33.5)	284 (66.5)	427 (100)	
Family size				
2–4	87 (29.8)	205 (70.2)	292 (68.4)	3.5; 0.29
> = 5	56 (41.5)	79 (58.5)	135 (31.6)	
Total	143 (33.5)	284 (66.5)	427 (100)	
Residence				
Urban	78 (24.8)	237 (75.2)	315 (73.8)	13.4; 0.0001
Rural	65 (58.0)	47 (42.0)	112 (26.2)	
Total	143 (33.5)	284 (66.5)	427 (100)	

#### Prevalence and risk factors of intestinal parasitosis

The overall prevalence of IP was 143 (33.5%). The prevalence of double and triple infection was 5.3 and 0.8%, respectively. According to our study, the identified risk factors for intestinal parasitosis were sex, age, water source, occupation and family size. The prevalence of IP was higher in males (35.7%) and in the age group of 15–19 (41.9%).

Intestinal parasitosis was also higher among farmers (41%), those who use well-dug water for drinking (50%) and those having relatively big family sizes (41.5%). *E. histolytica/dispar* (13.6%) followed by *G. lamblia* (7.7%) and *S. mansoni* (3.3%) were the most frequently identified parasite. *E. histolytica/dispar* was the most predominantly detected parasite (15.0%) followed by hookworm (4.2%) and *S. stercolaris* (2.8%) in males. In females, *S. mansoni* (4.7%) followed by *H. nana*, (4.2%) were the most common infections (Table 2). The proportion of *E. histolytica/dispar* (20%) was significantly higher in the age group of 15–19 years than others (P = 0.014). Moreover, the proportion of Hookworm infection was significantly higher in the age group of 35–39 years (19.0%) than others (P < 0.001) (Table 2).

**Table 2:** Distribution of intestinal parasites among outpatients at Adwa Health center, North Ethiopia, 2016

Variable	Type of parasite							
	*Eh* N (%)	*Gl* N (%)	*Sm* N (%)	Hw N (%)	*Ss* N (%)	*Hn* N (%)	*Ev* N (%)	Tsp. N (%)
Age in years								
15–19	32 (20.0)	16 (10.0)	9 (5.6)	3 (1.9)	–	6 (3.8)	2 (1.2)	–
20–24	7 (6.4)	8 (7.3)	3 (2.8)	1 (0.9)	3 (2.8)	4 (3.7)	–	–
25–29	3 (6.1)	5 (10.2)	1 (2.0)	2 (4.1)	2 (4.1)	1 (2.0)	–	–
30–34	4 (14.3)	1 (3.6)	1 (3.6)	2 (7.1)	1 (3.6)	1 (3.6)	–	1 (3.6)
35–39	3 (14.3)	1 (4.8)	–	4 (19.0)	2 (9.5)	–	–	–
> = 40	9 (15.0)	2 (3.3)	–	1 (1.7)	–	–	–	1 (1.7)
Total	58 (13.6)	33 (7.7)	14 (3.3)	13 (3.0)	8 (1.9)	13 (3.0)	2 (0.5)	2 (0.5)
P-value	0.014	0.53	0.32	< 0.001	0.27	0.65	0.65	0.1
Gender								
Male	32 (15.0)	18 (8.5)	4 (1.9)	9 (4.2)	6 (2.8)	4 (1.9)	–	2 (0.9)
Female	26 (12.1)	15 (7.0)	10 (4.7)	4 (1.9)	2 (0.9)	9 (4.2)	2 (0.9)	–
Total	58 (13.6)	33 (7.7)	14 (3.3)	13 (3.0)	8 (1.9)	13 (3.0)	2 (0.5)	2 (0.5)
P-value	0.47	0.87	0.59	0.16	0.15	0.25	0.16	0.16
Occupation								
Civil servant	17 (10.0)	14 (8.2)	2 (1.2)	3 (1.8)	1 (0.6)	3 (1.8)		2 (1.2)
Trader	2 (20.0)	–	1 (10.0)	1 (10.0)	1 (10.0)	1 (10.0)		
Farmer	34 (21.8)	15 (9.6)	5 (3.2)	7 (4.5)	5 (3.2)	4 (2.6)	1 (0.6)	
Housewife	5 (5.5)	4 (4.4)	7 (6.6)	2 (2.2)	1 (1.1)	5 (5.5)	1 (1.1)	
Total	58 (13.6)	33 (7.7)	14 (3.3)	13 (3.0)	8 (1.9)	13 (3.0)		2 (0.5)
P-value	0.002	0.38	0.19	0.23	0.39	0.14	0.63	0.39
Family size								
2–4	39 (13.4)	15 (5.1)	10 (3.4)	1 (3.4)	7 (2.4)	6 (2.1)	1 (0.3)	1 (0.3)
> = 5	19 (14.1)	18 (13.3)	4 (3.0)	3 (2.2)	1 (0.7)	6 (4.4)	1 (0.7)	1 (0.7)
Total	58 (13.6)	33 (7.7)	14 (3.3)	13 (3.0)	8 (1.9)	13 (3.0)	2 (0.5)	2 (0.5)
P-value	0.99	0.003	0.80	0.50	0.24	0.17	0.56	0.56
Source of drinking water								
Pipe	45 (12.9)	23 (6.6)	10 (2.9)	8 (2.3)	6 (1.7)	7 (2.0)	2 (0.6)	2 (0.6)
Spring	10 (15.4)	8 (12.3)	3 (4.6)	4 (6.2)	2 (3.1)	4 (6.2)	–	–
Dug-well	3 (21.4)	2 (14.3)	1 (7.1)	1 (7.1)	–	1 (7.1)	–	–
Total	58 (13.6)	33 (7.7)	14 (3.3)	13 (3.0)	8 (1.9)	13 (3.0)	2 (0.5)	2 (0.5)
P-value	0.02	0.19	0.55	0.26	0.66	0.11	0.80	0.80
Recurrent diarrhea								
Yes	3 (10.0)	2 (6.7)	2 (6.7)	4 (13.3)	–	3 (10.0)	–	–
No	55 (13.9)	31 (7.8)	12 (3.0)	9 (2.3)	8 (2.0)	10 (2.5)	2 (0.5)	2 (0.5)
Total	58 (13.6)	33 (7.7)	14 (3.3)	13 (3.0)	8 (1.9)	13 (3.0)	2 (0.5)	2 (0.5)
P-value	0.26	0.82	0.30	0.001	0.43	0.19	070	070

Eh, *E. histolytica*; Ss, *S. Stercolaris*

Gl, *G. lamblia*; Ev, *E. vermicularis*

Sm, *S. mansoni* Hn, *H. nana*

HW, hookworms; Tsp., taenia Spp

As shown in Table 2, the proportion of *E. histolytica* infection was significantly higher in males (P = 0.002) and in those who had dug-well as main water source for drinking than their counter parts (P = 0.02). Age was also significantly associated with hookworm infection (P < 0.0001). The proportion of *G. lamblia* infection was significantly higher among households with higher family size than their counter parts (P = 0.003). Recurrent diarrhea was also significant indicator of Hookworm infection (P = 0.013) (Table 2).

#### Intestinal parasitosis and haemoglobin level of study participants

The mean Hb concentration among the study participants was found to be 12.8 mg/dl. The overall prevalence of anaemia was 35 (8.2%). The mean haemoglobin level was 13.2 mg/dl) and 12.4 mg/dl for males and females, respectively. The highest prevalence of anaemia was recorded for the age group of 15–19 years (29.6%) (Table 3). The proportion of anaemia among intestinal parasite -infected and non-infected participants was 10.7 and 7.0%, respectively (presented in the Additional file 1).

The mean haemoglobin level was lower for patients infected with hookworms (11.04 mg/dl) and *S. stercoralis* (11.4 mg/dl) (P < 0.05).

Study participants infected with *S. stercoralis* were more likely to develop anaemia than the non- infected ones; AOR (adjusted odds ratio) = 5.3, 95% CI (1.01–27.4); P = 0.028. Likewise, patients infected with hookworm were more likely to develop anaemia than non-infected counter parts; AOR = 11.1, 95% CI (3.36–36.9); P = 0.000 (Table 3).

**Table 3:** Distribution of intestinal parasite species and anaemia at Adwa Health Center, North Ethiopia 2016

Parasite species	Mean (Std) Hb level (mg/dl)	Non-anemic (n = 392) (%)	Anemic (n = 35) (%)	P-value	AOR	95% CI
*Entameba histolytica*	12.94 ± 0.09	58 (14.8)	0	NA	NA	NA
*Giardia lamblia*	12.62 ± 0.02	29 (7.25)	4 (11.4)	0.15	2.22	0.71–6.9
*Schistosoma mansoni*	12.62 ± 0.06	13 (3.25)	2 (5.7)	0.89	1.14	0.14–9.1
*Enterobius vermicularis*	13.50 ± 0.01	2 (0.5)	0	NA	NA	NA
*Hymenolepis nana*	12.45 ± 0.08	2 (0.5)	0	NA	NA	NA
*Strongyloides stercolaris*	11.39 ± 0.08	6 (1.5)	3 (8.6)	0.028	5.3	1.01–27.4
Hookworms	11.04 ± 0.02	8 (2.0)	6 (17.1)	0.000	11.1	3.36–36.9
Taenia species	13.50 ± 0.02	2 (0.5)	–	0.71		

NA, not applicable

### Discussion

The prevailing prevalence of IP in the current study (33.5%.) was lower than results reported from Jimma, Ethiopia (83%) [6], Eastern Wollega (64.9%) [18], Northern Gondar (79.8%) [21], Southern Ethiopia (62.3%)[22] and Teda Health Center (62.3%) [23]. Moreover, higher prevalence was reported in Vietnam (88%) [19] and Nigeria (52%) [20]. This lower result in the current study might be due to the nature of study population in which school children were excluded.

In contrast, the present finding was higher than results from different localities of Ethiopia [2, 24, 25] and other parts of the world; Kashmir (18.02%) [26] and Saudi Arabia 6.2% [27]. This might be attributable to differences in environmental and personal hygiene, source of households’ water supply, and habit of walking bare-footed. The sensitivity of stool examination procedures might also play roles in the difference of IP prevalence.

The prevalence of amoebiasis (13.6%) in the present study was higher than studies from Jimma (5.6%) [6], Gondar (10.3%) [21], Saudi Arabia (4.7%) [27], Malaysia (0.4%) [28], Italy (4.1%) [29] and Myanmar (6.2%) [30]. The relatively higher prevalence of *E. histolytica/dispar* infection in this study might be due to poor access to safe drinking water supplies which is supported by our study that majority of the study participants used dung-well and spring water sources. Over diagnosis of *E. histolytica* is also expected from the current study since molecular based diagnostics were not used to differentiate *E. histolytica* from *E. dispar.*

The higher prevalence of hookworm infection in males than females in the present study was in agreement with studies conducted in certain parts of Ethiopia [2, 9, 21] and Brazil [39]. However, higher prevalence of hookworm infection in females was reported in Eastern Ethiopia [7], South Ethiopia [10] and Nepal [31]. The higher prevalence of hookworm in men in our study might be due to the fact that agrarian men are more frequently engaged in agricultural activities where shoe wearing is not convenient. In the present study, peak hookworm infection was recorded in participants of age 35–39 years. This result was in line with reports from Northwest Ethiopia [23], Southern Ethiopia [22], Uganda [15] and Nigeria [20, 32, 33]. Persistence of hookworms with age might be attributed to ignorance of foot wear among the adult agrarian males [20].

The significantly higher prevalence of *E. histolytica* infection among participants that used non-piped water supplies in the present study was consistent with reports from Eastern Wellega, Ethiopia [18]. This might be an indication for the incomplete separation of human waste with water sources that are used for drinking in the area. Our study also revealed that giardiasis was significantly higher among those with larger family sizes (> 5 members). Consistent findings were reported from Rural Malaysia [40] and Brazil [41, 42]. This might be due to occurrence of person-to-person transmissions through direct faecal-oral contact among family members with infected children.

Occupation was also found to be related to a risk of IP in our study. The prevalence of IP was significantly higher among farmers (41%). It was in agreement with findings from Ethiopia [42, 43] and Nigeria [44, 43]. This may be explained by the fact that farmers encounter constant contact with contaminated soil and water. Farmers also indiscriminately eat with unwashed hands after work.

In the present study the mean haemoglobin threshold was significantly lower in IP-infected than non-infected individuals (P = 0.002). Moreover, the proportion of anaemia was also higher among outpatients who harbored IPs. These were in agreement with several studies elsewhere [19, 34–37] in which intestinal parasites were strongly associated with development of anaemia.

In this study IP due to hookworm and *S. stercolaris* infections were significantly associated with anaemia. This conforms to other studies conducted in Mozambique [33], Uganda [15], Bangladesh [37], Nigeria [33], Cameroon [35], Vietnam [19] and Kenya [38]. This might be because Hookworm species ((*Ancylostoma duodenale* and *Necator americanus*) cause significant blood losses through feeding and cause a daily loss into the small intestine of 0.14–0.26 ml and 0.02–0.07 ml/worm, respectively) and oozing of the blood at attachment site [44].

### Conclusion

Co-existence of IP infections and anaemia is major public health significance among adolescents and adults in the study area. *E. histolytica* was significantly prevalent in the younger group (15–19 years) while hookworm infection persisted with age. Higher overall anaemia was observed for IP-infected patients.

## Limitations of the study

This study didn’t address the dietary and other factors that might contribute for occurrence of anaemia in the study participants.

## Additional file

**Additional file 1.** Questionnaire and laboratory data registration form. The questionnaire was intended to collect demographic data of study participants and factors associated with intestinal parasitosis. It includes sex, age, place of residence, occupation, family size and source of drinking water among others. The laboratory data registration form on the other hand was used to record results of stool examination and haemoglobin level.

## Abbreviations

Hb: haemoglobin
EDTA: ethylenediamine tetraacetic acid
IP: intestinal parasitosis
