# Supplementary material for: Intestinal parasitosis and anaemia among patients in a Health Center, North Ethiopia
Source: BMC Res Notes. 2017 Nov 28;10:632. doi: 10.1186/s13104-017-2957-2 (PMC5704372; doi:10.1186/s13104-017-2957-2)
Supplement: Supplementary file 1 — Additional file 1. Questionnaire and laboratory data registration form. The questionnaire was intended to collect demographic data of study participants and factors associated with intestinal parasitosis. It includes sex, age, place of residence, occupation, family size and source of drinking water among others. The laboratory data registration form on the other hand was used to record results of stool examination and haemoglobin level. [file 13104_2017_2957_MOESM1_ESM.docx]

# Mekelle University

# Department of Microbiology, Immunology and Parasitology

1. A Questionnaire of ‘**Intestinal parasitosis and anaemia among patients in a Health Center, North Ethiopia’.**

1. Questionnaire code ____________________

2. Name of interviewer ____________________

3. Date of interview (dd/mm/yyyy) ____________________

4. Age of study participant (years) ____________________

5. Sex of study participant a) Male b) Female

6. Place of residence a) Urban b) Rural

7. Family size a) < 5 b) > 5

8. Occupation a) Farmer b) Civil servant

c) Trader d) Housewife

9. Source of drinking water a) Pipe b) Spring c) Well-dug

10. History of recurrent diarrhea a) Yes b) No

# Laboratory data registration form

1. Stool examination result

a) Ova of parasites detected a) Yes b) No

b) Species of parasite identified Specify the species_________________

2. Hemoglobin value Specify hemoglobin level_________________

3. Anemia status a) Non-anemic b) Mild anemia

c) Moderate anemia d) Sever anemia
